# Strategies for the expansion of human induced pluripotent stem cells as aggregates in single-use Vertical-Wheel™ bioreactors

**DOI:** 10.1186/s13036-019-0204-1

**Published:** 2019-09-14

**Authors:** Diogo E. S. Nogueira, Carlos A. V. Rodrigues, Marta S. Carvalho, Cláudia C. Miranda, Yas Hashimura, Sunghoon Jung, Brian Lee, Joaquim M. S. Cabral

**Affiliations:** 1 Department of Bioengineering and iBB, Institute for Bioengineering and Biosciences, Instituto Superior Técnico, Universidade de Lisboa, Av. Rovisco Pais, 1049-001 Lisbon, Portugal; 2 The Discoveries Centre for Regenerative and Precision Medicine, Lisbon Campus, Instituto Superior Técnico, Universidade de Lisboa, Lisbon, Portugal; 3 PBS Biotech, Camarillo, CA USA

**Keywords:** Human induced pluripotent stem cells, Expansion, Aggregates, Fed-batch, Dextran sulfate, Single-use bioreactors, Vertical-Wheel bioreactors

## Abstract

**Background:**

Since their inception, human induced pluripotent stem cells (hiPSCs) have held much promise for pharmacological applications and cell-based therapies. However, their potential can only be realised if large numbers of cells can be produced reproducibly on-demand. While bioreactors are ideal systems for this task, due to providing agitation and control of the culture parameters, the common impeller geometries were not designed for the expansion of mammalian cells, potentially leading to sub-optimal results.

**Results:**

This work reports for the first time the usage of the novel Vertical-Wheel single-use bioreactors for the expansion of hiPSCs as floating aggregates. Cultures were performed in the PBS MINI 0.1 bioreactor with 60 mL of working volume. Two different culture media were tested, mTeSR1 and mTeSR3D, in a repeated batch or fed-batch mode, respectively, as well as dextran sulfate (DS) supplementation. mTeSR3D was shown to sustain hiPSC expansion, although with lower maximum cell density than mTeSR1. Dextran sulfate supplementation led to an increase in 97 and 106% in maximum cell number when using mTeSR1 or mTeSR3D, respectively. For supplemented media, mTeSR1 + DS allowed for a higher cell density to be obtained with one less day of culture. A maximum cell density of (2.3 ± 0.2) × 10^6^ cells∙mL^− 1^ and a volumetric productivity of (4.6 ± 0.3) × 10^5^ cells∙mL^− 1^∙d^− 1^ were obtained after 5 days with mTeSR1 + DS, resulting in aggregates with an average diameter of 346 ± 11 μm. The generated hiPSCs were analysed by flow cytometry and qRT-PCR and their differentiation potential was assayed, revealing the maintenance of their pluripotency after expansion.

**Conclusions:**

The results here described present the Vertical-Wheel bioreactor as a promising technology for hiPSC bioprocessing. The specific characteristics of this bioreactor, namely in terms of the innovative agitation mechanism, can make it an important system in the development of hiPSC-derived products under current Good Manufacturing Practices.

## Background

Human pluripotent stem cells (hPSCs), due to their ability to self-renew and to generate cells derived from the three germ layers, have a great potential for drug discovery, disease modelling and, ultimately, Regenerative Medicine applications [1, 2]. Human induced pluripotent stem cells (hiPSCs), first derived in 2007 from reprogramming of adult somatic cells [3, 4], are not only less ethically prohibitive than human embryonic stem cells (hESCs), but also open the way for personalised medicine approaches [5, 6].

One of the greatest constraints in using these cells, or their derivatives, for biomedical applications is their expansion to clinically-relevant numbers, which can reach 10^9^ cells per patient for Regenerative Medicine settings [7]. Traditional planar cell culture platforms, such as tissue culture plates, are unsuited for these large-scale applications [8]. Alternatively, bioreactors provide a 3D, scalable environment and are compatible with different medium feeding strategies, which can improve cell growth. In fact, planar culture platforms require a “repeated batch” strategy for medium change, where the medium is replaced at fixed time intervals, causing drastic variations in culture parameters such as pH or the concentration of nutrients, growth factors or metabolites. In contrast, bioreactors open the potential for alternative feeding strategies, such as fed-batch, where concentrated medium is added without replacing the contents of the culture vessel, or perfusion, where there is constant withdrawal of spent medium and replenishment of fresh medium [9, 10].

Early studies on the suspension culture of hPSCs as aggregates in bioreactors focused on the optimisation of the process [11–14] including the establishment of xeno-free conditions [15]. More recently, Kropp and colleagues [10] expanded hiPSCs in single-use, instrumented bioreactors, and were able to increase cell yield in 47% when applying a perfusion feeding strategy over repeated batch culture, obtaining a cell density of (2.85 ± 0.34) × 10^6^ cells∙mL^− 1^. These studies, however, were performed using common bioreactor systems, such as stirred tank bioreactors, which were developed and extensively used for manufacturing traditional biological products, where the quality of the cells is not the major concern. These bioreactors may not constitute the best solution for hPSC culture as they often require high agitations speeds in order to keep cell aggregates efficiently in suspension, with high shear rates conveyed to the cells by the impeller. As such, new bioreactor configurations are being developed to overcome these problems and allow for large-scale stem cell culture [16].

One example of a novel bioreactor system is the single-use Vertical-Wheel bioreactor (VWBR), available on a wide range of scales, from 100 mL to 80 L. The agitation in these bioreactors is provided by a large vertical impeller, which, combined with the U-shaped bottom, allows for a better homogenisation of the vessel contents with reduced power input. Consequently, cells are exposed to lower shear stress levels, when compared to traditional alternatives [17].

The VWBR has already been used for some stem cell-related applications, including for the growth of human mesenchymal stem cells [18–20] and, recently, it was proven to allow for the scalable expansion of hiPSCs on microcarriers in xeno-free conditions [21]. While microcarriers provide a surface for the cells to adhere to, thus easing transition from 2D cultures, the cells need to be detached at the end of the culture, increasing the load of the downstream processing and affecting cell quality. Culturing the cells as aggregates may provide a simpler and potentially more cost-effective process for large-scale hiPSC production [13, 22]. Moreover, with the VWBR, agitation can be used to control aggregate size, as large aggregates (> 300 μm) are subjected to diffusion problems to their centre, causing undue cell differentiation or death [23]. To minimise aggregate variability, however, there is a need for additional strategies for size control beyond physical forces. Polysulfated compounds are commonly used in the biopharmaceutical industry, not only for their ability to reduce cell aggregation by modulating charges in the surface of the cell, but also for their anti-apoptotic effect on cells [24, 25]. Dextran sulfate (DS), in particular, has been employed for various cell systems, such as insect [24] or Chinese Hamster Ovary cells [26]. Most notably, DS was recently reported to promote these effects on hPSCs without loss of pluripotency [27], thus, the use of this molecule in bioreactor systems may improve the outcomes in terms of cell expansion.

**Fig. 1:**
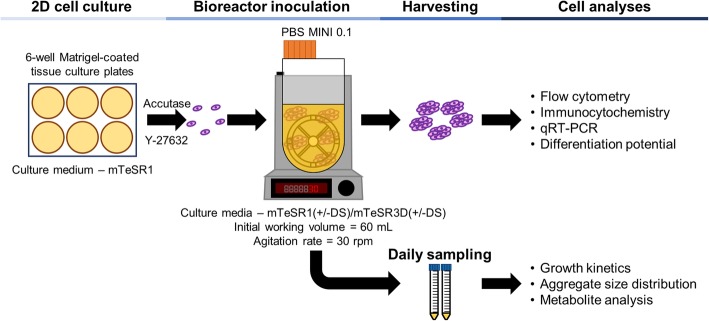
Workflow of the expansion of hiPSCs as aggregates in VWBRs. Following 2D culture in tissue culture plates, hiPSCs were dissociated into single cells with Accutase and inoculated into the PBS MINI 0.1 bioreactor, where they formed aggregates. The culture was evaluated through daily sampling and analysis of the cells after culture

In this work, hiPSCs were expanded for the first time as aggregates in VWBRs (PBS MINI 0.1), with a working volume of 60 mL (Fig. 1). The results were validated using a second cell line, evidencing the robustness of this culture system. In order to maximise the potential of this culture set-up, the novel VWBR was also combined with a fed-batch strategy, which was never before described for hiPSC culture, as well as with DS, which allowed to greatly increase the cellular growth in the reactor. The work here performed demonstrates that hiPSCs can be cultured as aggregates in the VWBRs, opening the path for current Good Manufacturing Practice (cGMP)-compliant protocols for expansion, and prospectively, differentiation of hiPSCs for clinical applications.

## Results

### VWBRs allow the expansion of hiPSCs as aggregates

A protocol for expansion of hiPSCs as aggregates in the VWBRs was adapted from similar reports for other types of bioreactors [10]. For initial experiments, mTeSR1 was selected as the culture medium, due to being a feeder-free and serum-free medium, which has already been well-documented for the expansion of hiPSCs [28, 29]. The PBS MINI 0.1 MAG bioreactor was used with an initial working volume of 60 mL, just enough to cover the vertical wheel, to ensure the optimised hydrodynamic profile of the VWBR [17]. Preliminary tests were performed to determine the minimum stirring speed, which leads to efficient suspension of the aggregates throughout the culture time. Stirring was thus set at 30 rpm since at lower speeds aggregate sedimentation in the bottom of the vessel was observed.

**Fig. 2:**
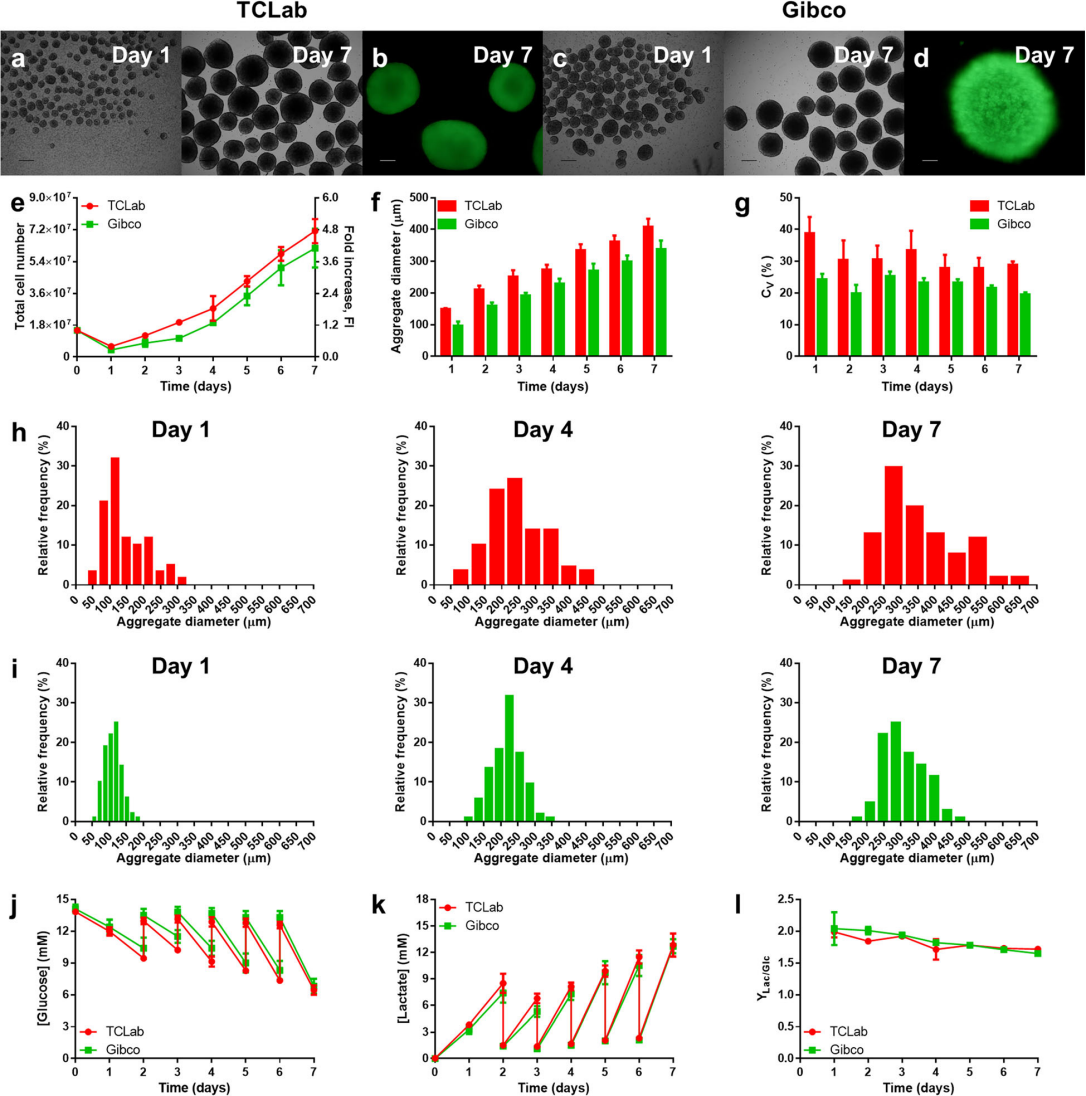
Results of the expansion of TCLab and Gibco hiPSCs with mTeSR1 in the PBS MINI 0.1 bioreactor. At day 0, the reactor was inoculated with 1.5 × 10^7^ cells (2.5 × 10^5^ cells∙mL^−1^) and 80% of the culture medium was changed daily starting from 48 h post-inoculation. **a-d** Representative images of cell aggregates harvested from the bioreactor, analysed through brightfield microscopy at days 1 and 7 of culture for **a** TCLab and **c** Gibco hiPSCs (scale bars = 250 μm) and through fluorescence microscopy at day 7 of culture culture for **b** TCLab and **d** Gibco hiPSCs following staining with calcein AM (scale bar = 100 μm). **e** Growth kinetics of the cells, in terms of total cell number and fold increase. **f-i** Aggregate size dynamics of the cells, in terms of **f** average aggregate diameter, **g** coefficient of variation, and aggregate size distribution profiles of a representative experiment at days 1, 4 and 7 post-inoculation for **h** TCLab and **i** Gibco hiPSCs. **j-l** Supernatant analysis of **j** glucose consumption and **k** lactate production, and **l** yield of lactate from glucose

For these initial experiments, two different cell lines were tested (TCLab and Gibco). In these culture conditions, the cells were able to form aggregates which grew throughout time (Fig. 2a, c) and were kept viable through 7 days of culture (Fig. 2b, d). For three independent bioreactor runs, the results were found to be reproducible, in terms of growth kinetics, aggregate size and metabolic profiles. The average growth curve obtained for this culture set-up is depicted in Fig. 2e. A maximum of (1.2 ± 0.1) × 10^6^ cells∙mL^− 1^ and (1.0 ± 0.2) × 10^6^ cells∙mL^− 1^ were obtained at day 7 post-inoculation with the TCLab and Gibco cell lines, respectively. The average diameter of cell aggregates was gradually increased to respective averages of 409 ± 25 μm and 338 ± 27 μm at day 7 (Fig. 2f). The dispersion of each experiment was measured through the coefficient of variation (Fig. 2g). For the TCLab cell line, the coefficient of variation was at its highest at day 1 (39 ± 5%), but generally decreased to ~ 30% in the last days of culture. For the Gibco cell line, it consistently remained below 25%. Aggregate diameter distributions at days 1, 4 and 7 are shown for a representative experiment in Fig. 2h, i. Culture medium supernatant analysis revealed that glucose levels were never depleted below 35% of fresh medium values (Fig. 2j), and lactate did not accumulate to concentrations over 16 mM (Fig. 2k). The yield of lactate from glucose remained between ~ 1.7 and ~ 2.1 throughout culture (Fig. 2l). These results overall show the implemented culture system to be robust, with similar results obtained for two different hiPSC lines.

### Fed-batch feeding sustains hiPSC growth but with lower cell expansion than repeated batch

Following the proof-of-concept experiments, demonstrating the use of the PBS MINI 0.1 for the expansion of two different hiPSC lines, different culture conditions were evaluated. In the previous experiments, mTeSR1 was used in repeated batch mode where, from day 2 onwards, 80% of the medium was changed daily. This approach results in significant variations in the culture parameters (e.g., glucose and lactate, as shown in Fig. 2g, h), which can have a negative effect on cell growth. However, while critical glucose and lactate concentrations were never reached with this feeding regime, daily medium changes are necessary in order to replenish components required to maintain the pluripotency of the cells, such as basic fibroblast growth factor (bFGF) and transforming growth factor β (TGFβ). mTeSR3D is an alternative formulation to mTeSR1, optimised for cell growth in suspension as the necessary growth factors are replenished through daily addition of a concentrated supplement. Thus, this fed-batch regime can allow for sustained maintenance of pluripotency factors, while causing less severe changes in other culture parameters.

**Fig. 3:**
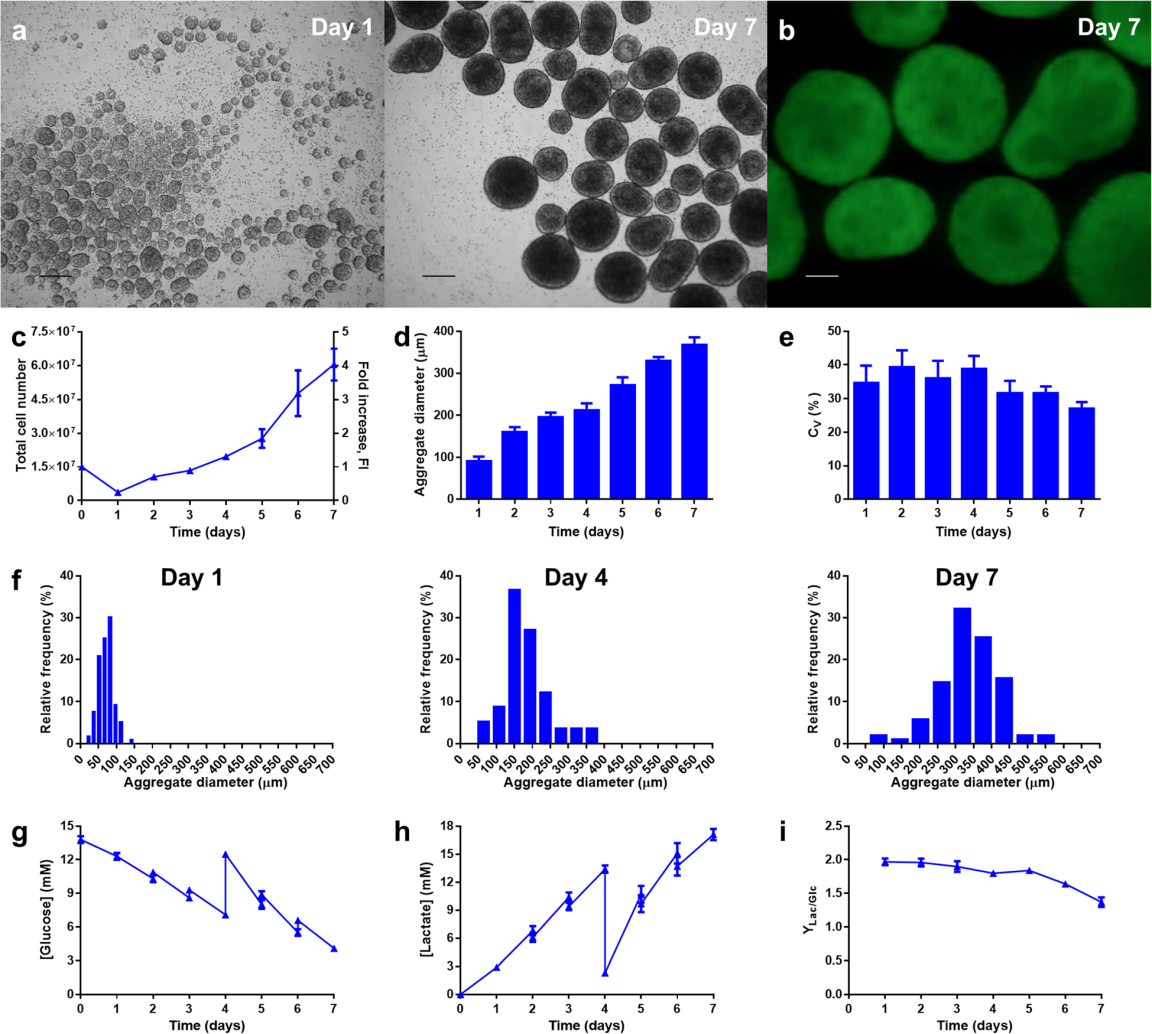
Results of the expansion of TCLab hiPSCs with mTeSR3D in the PBS MINI 0.1 bioreactor. At day 0, the reactor was inoculated with 1.5 × 10^7^ cells (2.5 × 10^5^ cells∙mL^− 1^) and 6.7 mL of feed medium was added daily starting from 48 h post-inoculation, with a full medium change at day 4. **a-b** Representative images of cell aggregates harvested from the bioreactor, analysed through **a** brightfield microscopy at days 1 and 7 of culture (scale bars = 250 μm) and **b** fluorescence microscopy at day 7 of culture following staining with calcein AM (scale bar = 100 μm). **c** Growth kinetics of the cells, in terms of total cell number and fold increase. **d-f** Aggregate size dynamics of the cells, in terms of **d** average aggregate diameter, **e** coefficient of variation, and **f** aggregate size distribution profiles of a representative experiment at days 1, 4 and 7 post-inoculation. **g-i** Supernatant analysis of **g** glucose consumption and **h** lactate production, and **i** yield of lactate from glucose

Aggregate formation with mTeSR3D was observed (Fig. 3a), and these aggregates remained viable until the end of culture (Fig. 3b). Despite the intrinsic advantages of this feeding regime, culture in mTeSR3D presented a similar, albeit lower, cell growth profile when compared to mTeSR1 (Fig. 3c), with a maximum of (8.8 ± 1.6) × 10^5^ cells∙mL^− 1^ at day 7. With mTeSR3D, aggregate diameter averaged 367 ± 18 μm at day 7 (Fig. 3d) with a coefficient of variation between ~ 25–40% (Fig. 3e) and following the distribution shown in Fig. 3f. These values were similar to those obtained with mTeSR1.

Expectedly, the fed-batch regime led to a more thorough consumption of glucose (Fig. 3g) without complete depletion (~ 50% prior to the medium change at day 4 and ~ 30% at day 7). Higher accumulation of lactate (Fig. 3h) was also observed, to a maximum of 19.3 mM at day 7, within the generally-considered inhibitory threshold for hPSCs of 15–20 mM [30, 31]. The yield of lactate from glucose (Fig. 3i) was maintained between ~ 1.8 and ~ 2.0 in the first 5 days of culture, indicating an essentially glycolytic metabolism. In fact, the growth of hiPSCs is sustained by rapid consumption of glucose via conversion to lactate (glycolysis) even in the presence of oxygen, in a phenomenon known as the Warburg effect [32, 33]. At days 6 and 7, the yield of lactate from glucose decreased to 1.64 ± 0.03 and 1.37 ± 0.07, suggesting an increasing shift to oxidative phosphorylation (OXPHOS). Nevertheless, this feeding regimen seems to be sufficient for the hiPSC maintenance, while minimising the variations in the culture environment to which the cells are subjected.

### Dextran sulfate supplementation improves cell yield

**Fig. 4:**
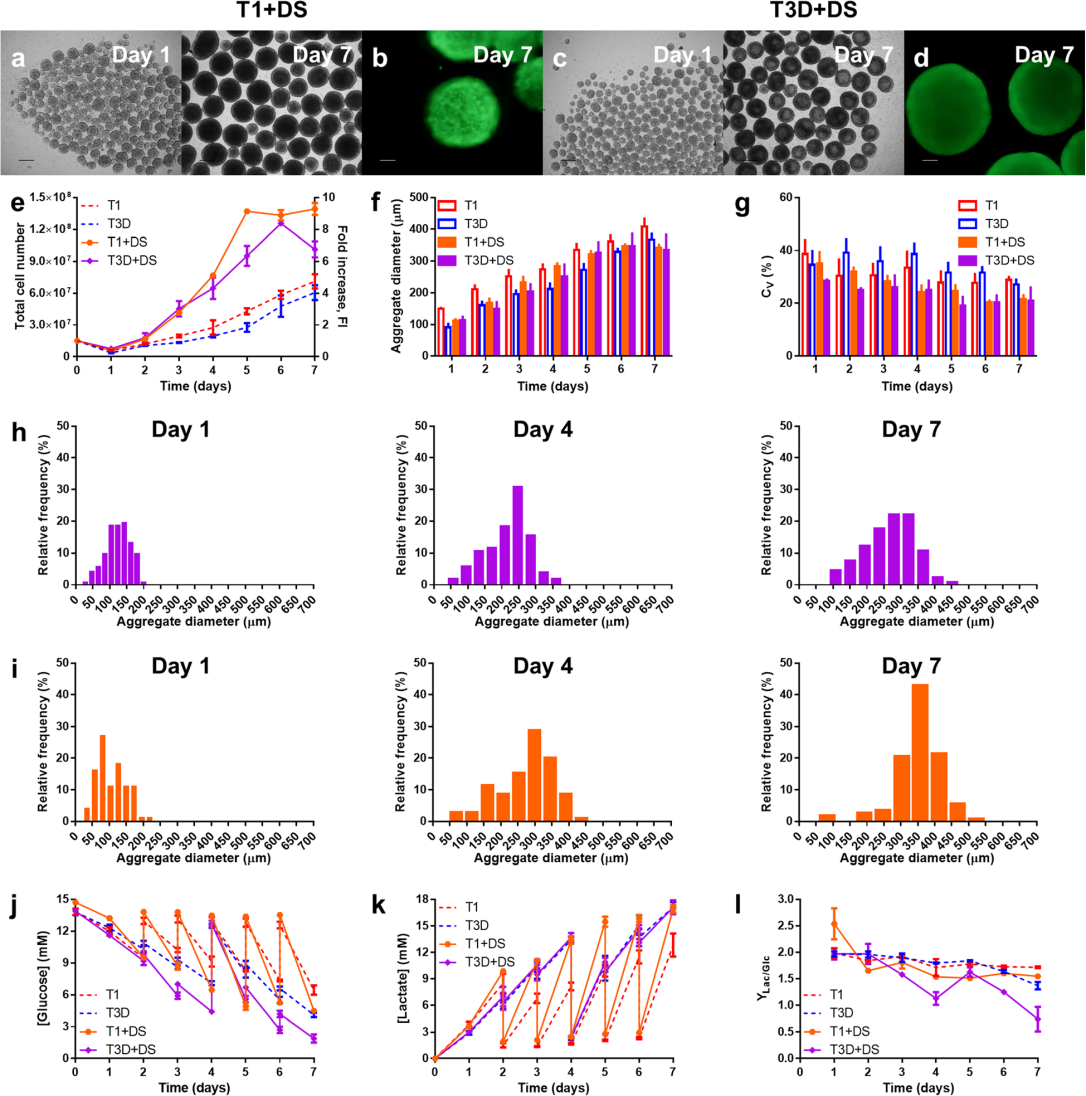
Results of the expansion of TCLab hiPSCs with mTeSR1 and mTeSR3D supplemented with dextran sulfate (T1 + DS and T3D + DS, respectively) in the PBS MINI 0.1 bioreactor. At day 0, the reactors were inoculated with 1.5 × 10^7^ cells (2.5 × 10^5^ cells∙mL^− 1^) and starting from 48 h post-inoculation, medium changes were perfomed according to the corresponding feeding regime. **a-d** Representative images of cell aggregates harvested from the bioreactor, analysed through brightfield microscopy at days 1 and 7 of culture in **a** T1 + DS and **c** T3D + DS (scale bars = 250 μm) and through fluorescence microscopy at day 7 of culture in **b** T1 + DS and **d** T3D + DS following staining with calcein AM (scale bars = 100 μm). **e** Growth kinetics of the cells, in terms of total cell number and fold increase. **f-i** Aggregate size dynamics of the cells, in terms of **f** average aggregate diameter, **g** coefficient of variation, and aggregate size distribution profiles of representative experiments at days 1, 4 and 7 post-inoculation for **h** T1 + DS and **i** T3D + DS. **j-l** Supernatant analysis of **j** glucose consumption and **k** lactate production, and **l** yield of lactate from glucose. mTeSR1 (T1) and mTeSR3D (T3D) data are shown for comparison

As an attempt to improve cell growth while reducing aggregate size and size variability, both culture media used in this study, mTeSR1 and mTeSR3D, were tested with DS supplementation in the VWBRs. DS-supplemented media allowed the hiPSCs to form aggregates and their viability at day 7 was not compromised (Fig. 4a-d). Growth curves (Fig. 4e and Additional file 1: Figure S1) show the hiPSCs to grow exponentially from 24 h of culture, reaching the maximum at day 5 for mTeSR1 + DS or day 6 with mTeSR3D + DS, following which the cell density stagnated or decreased, respectively. Supplementation with DS led to a higher maximum fold expansion with both media, 9.3 ± 0.6 ((2.3 ± 0.2) × 10^6^ cells∙mL^− 1^) for mTeSR1 + DS and 8.4 ± 0.1 ((1.79 ± 0.03) × 10^6^ cells∙mL^− 1^) for mTeSR3D + DS, representing a 97 and 106% increase in the number of cells versus the respective media without DS. Additionally, since the maximum in the number of cells is obtained 1 to 2 days earlier, these conditions also led to higher volumetric productivities of (4.6 ± 0.3) × 10^5^ cells∙mL^− 1^∙d^− 1^ and (2.99 ± 0.05) × 10^5^ cells∙mL^− 1^∙d^− 1^ for mTeSR1 + DS and mTeSR3D + DS, respectively, having increased 170 and 149% from non-supplemented media.

The maximum aggregate sizes obtained at the plateau were 346 ± 11 μm and 347 ± 39 μm, respectively (Fig. 4f), with coefficients of variation of about 20% (Fig. 4g). Interestingly, the effect of DS on aggregate size was not significant during the exponential growth phase of culture. Individual aggregate diameters are also similar between both supplemented media, following comparable distributions for all culture days (Fig. 4h, i).

Considerable glucose depletion was found for both media, with as little as 28 and 8% of fresh medium values at day 7 for mTeSR1 + DS and mTeSR3D + DS, respectively (Fig. 4j), and lactate was built-up until nearly 20 mM (Fig. 4k). For mTeSR1 + DS, following a yield of lactate from glucose of ~ 2.5 at day 1, it stabilised between 1.5 and 1.6 towards the end of culture. For mTeSR3D + DS, this yield was maintained at ~ 2.0 for the initial days of culture, but decreased substantially starting from day 3, reaching a minimum of ~ 0.7 (Fig. 4l). This value indicates the metabolism to, most likely, have shifted to being predominantly OXPHOS, in particular with mTeSR3D + DS.

### VWBRs do not compromise the pluripotency of the cells

**Fig. 5:**
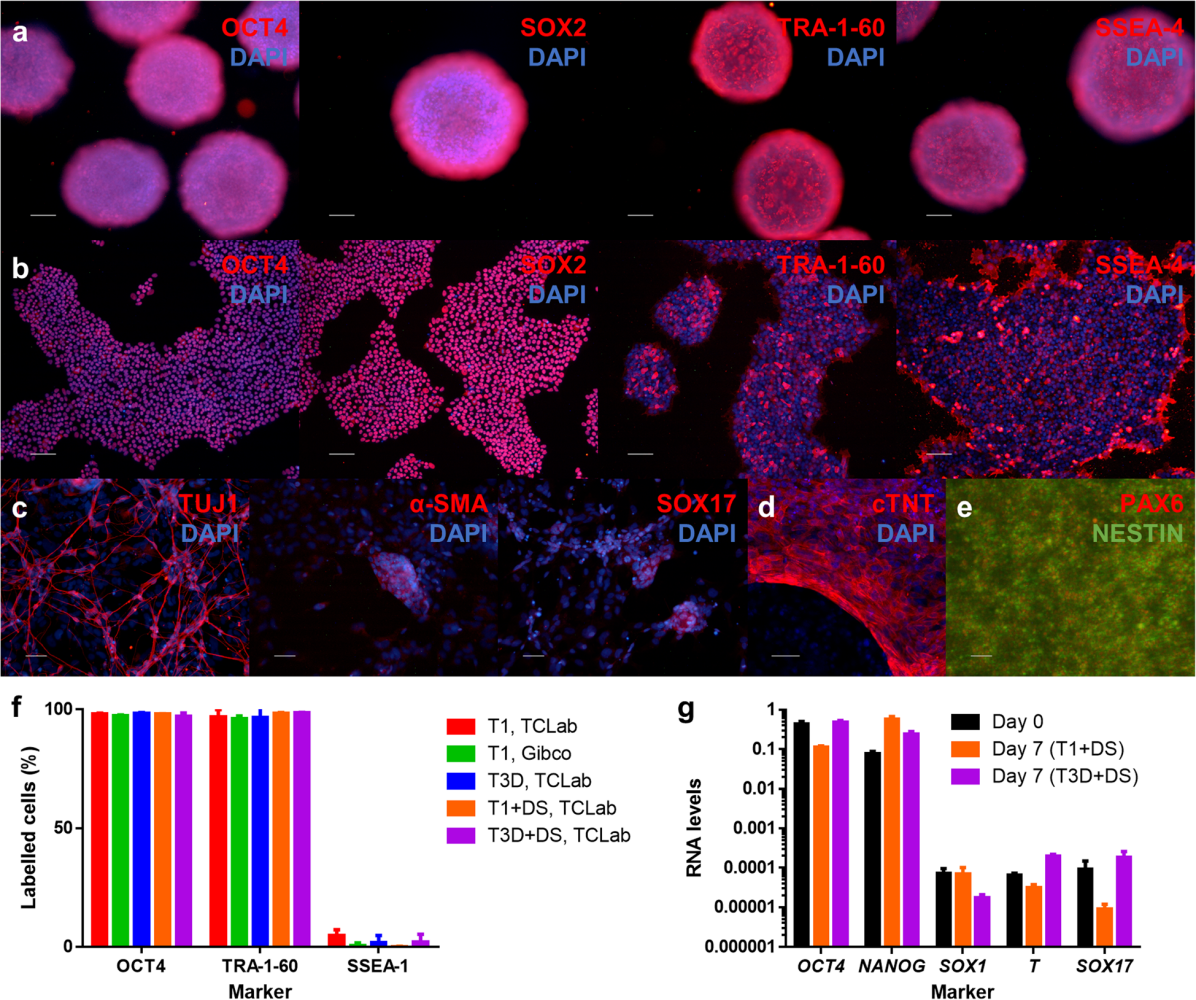
Characterisation of the hiPSCs following expansion in the PBS MINI 0.1 bioreactor. **a-b** Immunocytochemistry staining for pluripotency markers OCT4, SOX2, TRA-1-60 and SSEA-4 of cell aggregates harvested from the PBS MINI 0.1 bioreactor at day 7 post-expansion (**a**; scale bars = 100 μm), and of cells which were dissociated with Accutase and replated on 2D tissue culture plates (**b**; scale bars = 100 μm). **c** Immunocytochemistry staining of cells harvested from the bioreactor and left to form EBs for 5 weeks. The cells were stained for germ layer markers TUJ1 (ectoderm), α-SMA (mesoderm) and SOX17 (endoderm; scale bars = 50 μm). **d-e** Immunocytochemistry staining of cells harvested from the bioreactor and differentiated to **d** cardiomyocytes and **e** neural progenitors (scale bars = 50 μm). Cardiomyocytes were stained for cTNT, while neural progenitors were stained for PAX6 and NESTIN. **f** Flow cytometry analysis of cells harvested from the bioreactor after 7 days of expansion in the PBS MINI 0.1 bioreactor. The cells were labelled for pluripotency (OCT4 and TRA-1-60) and early differentiation (SSEA-1) markers. **g** qRT-PCR analysis of cells prior to (day 0) and following (day 7) expansion in the PBS MINI 0.1 bioreactor. The cells were tested for pluripotency (*OCT4* and *NANOG*) and germ layer (*SOX1*, *T/BRACHYURY* and *SOX17*, representing ectoderm, mesoderm and endoderm, respectively) marker expression. RNA levels are relative to expression of *GAPDH* and were computed as 2^–ΔCT^

Although the main purpose of expansion in a bioreactor system is to obtain large numbers of cells, it is important to guarantee that the process does not compromise cell quality, in particular hiPSC pluripotency. For that purpose, hiPSC aggregates were harvested from the PBS MINI 0.1 after culture and stained for both intracellular (OCT4 and SOX2) and surface (TRA-1-60 and SSEA-4) pluripotency markers. Figure 5a shows representative images, demonstrating the presence of these markers after 7 days of expansion. Cell aggregates were also dissociated into single cells with Accutase and replated on Matrigel-coated 2D tissue culture plates. These cells were able to form hiPSC colonies in these conditions and representative images show expression of the aforementioned markers (Fig. 5b). Assessment of the differentiation potential of the expanded cells was performed via the embryoid body (EB) assay (Fig. 5c), where the cells stained for markers of the three germ layers – TUJ1 (ectoderm), α-SMA (mesoderm) and SOX17 (endoderm), after 5 weeks of spontaneous differentiation. Expanded hiPSCs were also shown to be able to form both cardiomyocytes (Fig. 5d) and neural progenitors (Fig. 5e) following directed differentiation in 2D.

Quantification of pluripotency and differentiation marker expression was performed through both flow cytometry and quantitative real-time polymerase chain reaction (qRT-PCR) analyses. Flow cytometry analyses (Fig. 5f and Additional file 1: Figure S2) revealed that, for all conditions, following 7 days of expansion in the VWBR, expression of pluripotency markers OCT4 and TRA-1-60 was always above 90% and expression of early differentiation surface marker SSEA-1 was maintained at less than 10%. Before and after expansion, total RNA was extracted from cell samples and the expression of pluripotency (*OCT4* and *NANOG*) and differentiation genes, namely *SOX1* (ectoderm), *T/BRACHYURY* (mesoderm) and *SOX17* (endoderm), were assayed through qRT-PCR (Fig. 5g). Expression of pluripotency genes *OCT4* and *NANOG* was high and not significantly different between days 0 and 7, while the expression of the differentiation markers was maintained low. In general, all these results point out the VWBR not to compromise the pluripotency of the cells throughout the expansion process.

## Discussion

Therapeutic or pharmacological applications of hiPSCs require high numbers of cells. High cell densities of hPSCs have been previously attained using spinner flasks and stirred tank bioreactors, both using microcarriers as a culture support, or growing the cells as self-forming aggregates. However, some characteristics of these reactors, namely the low efficiency to keep in suspension particles such as cell-loaded microcarriers or cell aggregates, or the consequent high shear stress conveyed to the cells by the impeller at high stirring speeds, have led to research on more suitable bioreactor configurations for hPSC growth.

The work here described is intended to establish, in the PBS MINI VWBR, the culture of hiPSCs as floating aggregates. The largest barrier for the usage of this culture format is the aggregate size control [23]. Since in bioreactors aggregate size is influenced by shear stress [34], the VWBR is expected to provide a significant advantage, as its novel agitation mechanism leads to a more homogeneous shear stress distribution than observed in stirred tank bioreactors [17], contributing to a decrease in aggregate size variability and avoiding the formation of very large aggregates.

An overview of the results, already described in the previous section, is shown in Table 1. Initial experiments with the VWBR have shown it to allow for the growth of hiPSCs with mTeSR1, with high reproducibility between different bioreactor runs and among two cell lines (Fig. 2). Cell density values and volumetric productivities were also amongst those reported in spinner flasks and traditional reactors (Table 2). Culture performance can also be favourably compared with hiPSC culture on microcarriers in the VWBR [21], where similar cell densities and volumetric productivities were obtained with the same cell line. Despite this, the culture set-up is barely optimised, as around 60% of the cells did not aggregate in the first 24 h of culture and therefore further optimisation should be possible to improve the present results.

**Table 1:** Main results for all different tested conditions

Cell line	Culture medium	Fold change in cell number (day 1)	Maximum number of cells (cells/vessel)	Maximum cell density (cells∙mL^− 1^)	Maximum fold increase	Maximum specific growth rate (d^− 1^)	Minimum doubling time (d)	Volumetric productivity (cells∙mL^− 1^∙d^− 1^)	Maximum aggregate diameter (μm)	Minimum coefficient of variation
TCLab	mTeSR1	0.40 ± 0.03	(7.1 ± 0.7) × 10^7^	(1.2 ± 0.1) × 10^6^	4.8 ± 0.5	0.69 ± 0.13	1.1 ± 0.2	(1.7 ± 0.1) × 10^5^	409 ± 25	28 ± 6%
Gibco	mTeSR1	0.27 ± 0.08	(6.2 ± 1.1) × 10^7^	(1.0 ± 0.2) × 10^6^	4.1 ± 0.7	0.66 ± 0.05	1.06 ± 0.09	(1.4 ± 0.3) × 10^5^	338 ± 27	19.6 ± 0.6%
TCLab	mTeSR3D	0.24 ± 0.03	(6.1 ± 0.7) × 10^7^	(8.8 ± 1.6) × 10^5^	4.0 ± 0.5	1.1 ± 0.1	0.63 ± 0.06	(1.2 ± 0.1) × 10^5^	367 ± 18	27 ± 2%
TCLab	mTeSR1 + DS	0.38 ± 0.06	(1.4 ± 0.1) × 10^8^	(2.3 ± 0.2) × 10^6^	9.3 ± 0.6	1.1 ± 0.2	0.64 ± 0.11	(4.6 ± 0.3) × 10^5^	346 ± 11	20 ± 1%
TCLab	mTeSR3D + DS	0.50 ± 0.07	(1.26 ± 0.02) × 10^8^	(1.79 ± 0.03) × 10^6^	8.4 ± 0.1	0.96 ± 0.10	0.74 ± 0.09	(2.99 ± 0.05) × 10^5^	347 ± 39	19 ± 2%

**Table 2:** Comparison of aggregate-based suspension culture set-ups for the expansion of hPSCs

Cell type	Culture medium	Bioreactor type/volume	Inoculum cell density (cells∙mL^−1^)	Days in culture	Maximum cell density (cells∙mL^− 1^)	Volumetric productivity (cells∙mL^− 1^∙d^− 1^)	Reference
hESCs	mTeSR1	Spinner flask (100 mL)	1.8 × 10^4^	6	4.5 × 10^5^	7.5 × 10^4^	[11]
hESCs	mTeSR1	Spinner flask (50 mL)	1 × 10^6^	7	2.4 × 10^6^	3.4 × 10^5^	[12]
hESCs	StemPro hESC SFM	Spinner flask (60 mL)	2.5 × 10^5^	3–4	(1.1 ± 0.1) × 10^6^	(3.7 ± 0.3) × 10^5^	[13]
hiPSCs	E8	Spinner flask (45 mL)	(4-5)×10^5^	3–4	(1.8 ± 0.3) × 10^6^	(5.8 ± 0.8) × 10^5^	[14]
hESCs	mTeSR1	Spinner flask (100 mL)	2 × 10^4^	6	(2.4 ± 0.09) × 10^5^	(4.0 ± 0.2) × 10^4^	[15]
hiPSCs	mTeSR1/E8	Stirred tank (125 mL)	5 × 10^5^	7	(2.85 ± 0.34) × 10^6^	(4.07 ± 0.49) × 10^5^	[10]
hiPSCs	mTeSR1	Vertical-Wheel (60 mL)	2.5 × 10^5^	7	(1.2 ± 0.1) × 10^6^	(1.7 ± 0.1) × 10^5^	This study
hiPSCs	mTeSR1 + DS	Vertical-Wheel (60 mL)	2.5 × 10^5^	7	(2.3 ± 0.2) × 10^6^	(4.6 ± 0.3) × 10^5^	This study

Besides the promising results obtained with mTeSR1, a fed-batch feeding regime with mTeSR3D medium was tested as a means of mitigating the drastic parameter variations characteristic of a repeated batch approach, and allowing for the accumulation of growth-inducing paracrine factors [10]. The fed-batch strategy (Fig. 3) resulted in similar growth profiles and aggregate diameter distributions to repeated batch cultures but the high cell densities at the end of culture along with the slow replenishment of glucose and low dilution of lactate may have led to alterations in the metabolism of the cells. Proliferative cells, such as hiPSCs during their exponential growth, favour glycolysis for fast glucose uptake and to produce all of the molecules required for cell division [32, 33]. In fact, during the early days of culture, cells were metabolising glucose mainly through glycolysis, indicated by a yield of lactate from glucose close to 2. The lower yield observed at the end of culture suggests a metabolic shift to OXPHOS, which has already been reported in stirred bioreactor cultures of hiPSC [10]. Still, this is the first successful report indicating the viability of a fed-batch feeding strategy for the dynamic suspension culture of hiPSCs, and its optimisation could greatly increase the performance and the economic viability of this regime. A rough cost estimation based on these results led to the conclusion that mTeSR3D can provide about 10% reduction in the cost of medium spent per cell obtained. As such, the selection of the optimal medium for culture should be weighted considering the importance of both the cost and the performance.

The VWBR was shown to sustain the growth of hiPSCs with two different feeding regimes and to compare favourably with traditional bioreactor systems. However, given that the maximum cell density obtained was (1.2 ± 0.1) × 10^6^ cells∙mL^− 1^ and that Regenerative Medicine applications can require 10^9^ cells, it was considered important to develop strategies to increase the cell yield in the reactor. Additionally, the average size of the aggregates in all conditions exceeded 300 μm, which is problematic due to the diffusion limitations to the centre of the aggregate. A promising strategy that was found for both increased cell yield and aggregate size control was the supplementation of the culture media with DS. This compound was already reported to have these effects on hPSC aggregates [27] due to its anti-apoptotic activity and surface charge modulation, without compromising pluripotency. In fact, DS supplementation increased cell yields in about two-fold and reduced the period of exponential growth, allowing for faster cultures (Fig. 4). At day 1, we did not observe a reduction in aggregate sizes with DS supplementation (Fig. 4f) but our results suggest that a higher absolute number of aggregates was formed in the presence of DS (Additional file 1: Figure S3). In this regard, we may hypothesize that the increase in cell number resulted from an initial formation of a higher number of aggregates that expanded to a comparable size of those cultured in the non-supplemented media.

The results obtained thus clearly favour DS supplementation of media over its absence, due to the boost in culture performance without any observed negative effect over the hiPSCs. In these conditions, the fed-batch system had a lower overall performance when compared to mTeSR1, in terms of maximum cell yield, productivity and even in the effect on the metabolic activity of the cells.

It is important to note that the pluripotency and viability of the cells cultured under the optimal condition, mTeSR1 + DS, was not affected in the VWBR (Fig. 5). Indeed, after 7 days of culture, cells continued to express pluripotency markers, were able to form EBs, containing representatives of the three germ layers and to generate both cardiomyocytes and neural progenitors following directed differentiation into these lineages. At the end of the culture, cell aggregates reached over 300 μm in diameter, indicating some of the larger aggregates to be prone to diffusional limitations of nutrients and oxygen. While this could translate to necrosis in the centre of the aggregate, calcein AM/ethidium homodimer staining and confocal microscopy analysis as well as LIVE/DEAD flow cytometry (Additional file 1: Figure S4) show that dead cells are present in minority, and that there is no clear dead mass in the centre of the aggregate. Nevertheless, more prolonged culture of these cells would likely require dissociation and passaging to new culture vessels in order to maintain their viability and pluripotency over longer periods of time. Increasing the agitation at later days of culture could also reduce the diameter of the aggregates, allowing for better diffusion, while also contributing to a reduction in the variation in diameter size.

This study shows that VWBR can be considered a viable alternative for the growth of hPSCs, generating cell densities well within the range of those obtained with other types of bioreactors (Table 2), while maintaining pluripotency. Nevertheless, there are still some challenges to tackle with this culture set-up. Namely, the process is still ill-optimised and a design of experiments (DoE) approach could be employed as a means of maximising the cellular growth and controlling cell aggregation for prospective integrated differentiation approaches inside the bioreactor. The cell yield obtained in this study was 2.3 × 10^6^ cells∙mL^− 1^, after 5 days, using a repeated batch feeding strategy. Kropp and co-workers [9] described a 47% increase in cell yield when the repeated batch feeding was replaced by perfusion, in miniaturised bioreactor systems, allowing for a maximum cell density of (2.85 ± 0.34) × 10^6^ cells∙mL^− 1^ following 7 days in culture. Herein, the feeding modes used led to glucose depletion and lactate accumulation at later days of culture, especially in the fed-batch culture with mTeSR3D + DS, which may have hindered the achievement of higher cell densities. Operation of the PBS MINI 0.1 VWBR with perfusion is not straightforward but its implementation (either in this model or, prospectively, in higher volume models) would probably allow for higher cell densities to be obtained. Nevertheless, the cell density obtained with the present culture methodology was very close to that obtained by Kropp and co-workers with perfusion and, moreover, in comparison with that same study, our process started from half the cell density and reached the maximum cell density in less time (5 days vs 7 days).

The PBS MINI 0.1 is a scaled-down bioreactor model, designed for process development and optimisation and not for cell production for clinical/industrial use. Assuming that 10^9^ cells are required for organ regeneration (e.g. heart or liver [7]), if the highest cell density obtained in this study, (2.3 ± 0.2) × 10^6^ cells∙mL^− 1^, could be maintained at higher scales, a bioreactor with a working volume of about 450 mL would be enough to meet the needs for one person. The availability of VWBR up to 80 L allow to envisage the use of higher volume models to generate cells for allogeneic settings while smaller-scale vessels (e.g. PBS 3), could be suited for an autologous cell product. Independently of the application, it is important to note that the scale-up of hPSCs is often prohibitive due to medium costs and, as such, it is crucial to carefully plan how to perform it, namely in terms of which criteria to apply (constant power input per volume, tip speed, mixing time or oxygen transfer, for instance). A previous study [21] already proved the PBS MINI 0.5, which can hold up to 500 mL, to be compatible with the growth of hiPSCs attached to microcarriers. As such, it would be important to also scale-up cell growth as aggregates, in order to generate a number of cells compatible with clinical and/or pharmacological applications.

## Conclusions

One of the main bottlenecks in the usage of hPSCs in clinical or pharmacological applications is their expansion to large quantities, while maintaining their characteristics. Bioreactors provide major advantages over planar culture platforms, namely in terms of scalability, control and homogeneity, but are still not fully optimised for stem cell growth, as the shear stress caused by the impeller can greatly damage these cells. The VWBRs can mitigate this problem due to their more efficient mixing and gentler agitation set-up. The work here described is one of the first accounts of the usage of the PBS MINI VWBR for hPSC expansion, and the first one to expand the cells as floating aggregates. This system overall has the potential to comply easily with cGMP due to the single-use bioreactor system and the lack of matrices of any kind for cell adherence. The conjunction of mTeSR1 medium with DS led to a maximum increase of 9.3 ± 0.6-fold over the inoculum after only 5 days of culture, while not compromising the pluripotency of the cells. Although there are still some challenges to face with this system, namely the usage of xeno-free media, the conversion to perfusion in order to potentially generate larger numbers of cells, and, finally, the scale-up to larger bioreactors, this study provides compelling evidence in the applicability of the VWBRs for the growth of hPSCs for diverse biomedical applications.

## Methods

### Human induced pluripotent stem cell culture and maintenance

This work was performed using two different hiPSC lines. The F002.1A.13 cell line, (TCLab – Tecnologias Celulares para Aplicação Médica, Portugal), referred to in the text as “TCLab”, was reprogrammed from human healthy fibroblasts (46, XX), through retroviral transduction of human genes *OCT4*, *SOX2*, *C-MYC* and *KLF4*. The Gibco® human episomal induced pluripotent stem cell line (Thermo Fisher Scientific, USA), referred to in the text as “Gibco”, was derived from CD34^+^ cord blood through EBNA-based episomal transfection of factors SOX2, OCT4, KLF4, C-MYC, NANOG, LIN28 and SV40 T antigen. The hiPSCs were cultured on 6-well tissue culture plates coated with Matrigel (1:100; Corning, USA), in mTeSR1 culture medium (STEMCELL Technologies, Canada), and kept in a humidified incubator at 37 °C and 5% CO_2_. Culture medium was refreshed daily, and the cells were routinely passaged after reaching 80% confluence at a split ratio of 1:4, using EDTA (Thermo Fisher Scientific) [35]. Briefly, cells were washed twice and left to incubate for 5 min with EDTA (0.5 mmol∙L^− 1^ in Dulbecco’s phosphate-buffered saline, DPBS; Thermo Fisher Scientific). Afterwards, EDTA was removed and the cells were rinsed and collected by pipetting with culture medium before plating in new Matrigel-coated tissue culture plates. Cultures did not exceed four passages prior to bioreactor inoculation.

### Bioreactor inoculation and operation

For this work, the PBS MINI 0.1 MAG VWBRs, which hold a maximum volume of 100 mL, were used. The working volume was selected to allow complete covering of the impeller wheel (initial working volume = 60 mL). Cells from 80% confluent 6-well tissue culture plates were incubated for 1 h in mTeSR1 supplemented with 10 μmol∙L^− 1^ ROCK inhibitor Y-27632 (STEMCELL Technologies) prior to harvesting with Accutase. Briefly, cells were washed once with DPBS and incubated for 7 min at 37 °C in Accutase (Sigma, USA). The cells were harvested and mechanically dissociated into single cells with a micropipette, and diluted with culture medium, following centrifugation at 210×*g* for 3 min and resuspension in culture medium (mTeSR1 or mTeSR3D, STEMCELL Technologies) supplemented with Y-27632. The hiPSCs were counted with a haemocytometer, using the trypan blue dye exclusion test, and seeded in the bioreactor at a density of 250,000 cells∙mL^− 1^. Culture media with Y-27632 was added until reaching the working volume. For culture in mTeSR1, the medium was changed after 48 h to mTeSR1 without Y-27632, and from then on, 80% of the volume was changed daily. For culture in mTeSR3D, cells were initially cultured in seed medium, and, starting from 48 h post-inoculation, 6.7 mL of feed medium were added daily. At day 4, the medium was replaced with fresh seed medium, and from then on, 6.7 mL of feed medium were once again added daily until the end of culture. When used, DS (Sigma) was supplemented only on day 0 at a concentration of 100 μg∙mL^− 1^ [27]. Bioreactor cultures were maintained for 7 days and the stirring was continuously maintained at 30 rpm to keep the aggregates in suspension.

Culture sampling was performed daily. Two samples of 700 μL were collected with the reactor under agitation, and photos of the aggregates were captured with an inverted optical microscope (Leica DMI3000B/Nikon Digital Camera Dxm1200F) for later measurement. At least 50 aggregates were captured and analysed per timepoint. The area of the aggregates in each photo was determined using the FIJI software [36, 37], and their diameter was computed, considering the aggregates to be approximately spherical, as

1 $$ d=\sqrt{\frac{4A}{\pi }} $$

with A as the area of the aggregate. The dispersion in aggregate size was determined as the coefficient of variation, defined as

2 $$ {C}_V=\frac{{\mathrm{SD}}_d}{\mu_d} $$

with SD_d_ as the standard deviation and μ_d_ as the average of the diameter for each condition. The aggregates were incubated with Accutase and mechanically dissociated into single cells as previously described. Viable cells were counted with a haemocytometer. Cell viability was over 90% at all culture days. Fold increase in cell number at a given time point was calculated as the ratio

3 $$ \mathrm{FI}=\frac{X}{X_0} $$

with X as the number of cells at the considered time point, and X_0_ as the initial number of cells.

The viability of cells in the aggregates was assayed by incubation with 2 μmol∙mL^− 1^ of calcein AM during 20 min and observation of the stained aggregates under a fluorescence microscope (Leica DMI3000B/Nikon Digital Camera Dxm1200F).

At the end of culture, hiPSC aggregates were incubated for 1 h in culture medium supplemented with Y-27632 following incubation with Accutase and mechanical dissociation into single cells, as previously described, and replating on Matrigel-coated tissue culture plates at a density of 5 × 10^4^ cells∙cm^− 2^.

### Glucose and lactate analysis

Culture supernatants were collected every day prior to and following medium exchange, and centrifuged at 360×*g* for 10 min to remove dead cells and debris. The cell-free supernatants were analysed using an YSI 7100MBS Multi Channel Biochemistry Analyser (Yellow Spring Instruments, USA) for concentrations of glucose and lactate. The apparent yield of lactate from glucose was calculated for each day as

4 $$ {Y}_{\mathrm{Lac}/\mathrm{Glc}}=\frac{\Delta  \mathrm{Lac}}{\Delta  \mathrm{Glc}} $$

with ΔLac as the production of lactate and ΔGlc as the consumption of glucose during a given day of culture.

### Flow cytometry

Throughout culture, cells were collected from the PBS MINI 0.1 and analysed for the expression of pluripotency and differentiation markers. The protocols for staining are described elsewhere for both intracellular [38] and surface markers [21]. For intracellular staining, an antibody for OCT4 (1:300; Millipore, USA) was used, and goat anti-mouse IgG-AlexaFluor 488 (1:300; Thermo Fisher Scientific) was used both as a secondary antibody and as a negative control. The gate was selected to contain only 1% of false positives (i.e. 1% of the negative control samples). For surface staining, cells were labelled with antibodies for TRA-1-60 (1:10, PE-conjugated; Miltenyi Biotec, Germany) and SSEA-1 (1:20; FITC-conjugated, BioLegend, USA). The gate was selected to contain only 1% of false positives (i.e. 1% of the unstained samples). The cell samples were analysed with a FACSCalibur flow cytometer (Becton Dickinson, USA) and acquisition of the data was performed with the Cell Quest software (Becton Dickinson). For data analysis, Flowing Software (University of Turku, Finland) was used. A minimum of 10,000 events were analysed for each sample.

### Immunocytochemistry

Both replated cells and aggregates were stained using previously described protocols for both intracellular [38] and surface markers [21]. For intracellular staining of hiPSCs, antibodies for OCT4 (1:150; Millipore) and SOX2 (1:200; R&D Systems, USA) were used, and goat anti-mouse IgG-AlexaFluor 546 (1:500; Thermo Fisher Scientific) was used as a secondary antibody. For surface staining of hiPSCs, cells were labelled with antibodies for TRA-1-60 (1:135, StemGent, USA) and SSEA-4 (1:135; StemGent) with the secondary antibody goat anti-mouse IgM-AlexaFluor 546 (1:500; Thermo Fisher Scientific) or goat anti-mouse IgG-AlexaFluor 546 (1:500), respectively. Staining of EBs was performed with antibodies for TUJ1 (1:1000; Covance, USA), α-SMA (1:200; Dako, Denmark), and SOX17 (1:100; R&D Systems). Staining following directed differentiation was performed with cTNT (1:200; Thermo Fisher Scientific) for cardiac differentiation, and PAX6 (1:400; Covance) and NESTIN (1:400; R&D Systems) for neural differentiation. Secondary staining was performed with goat anti-mouse IgG-AlexaFluor 546 (1:500), goat anti-rabbit IgG-AlexaFluor 546 (1:500, Thermo Fisher Scientific) or goat anti-mouse IgG-AlexaFluor 488 (1:500; Thermo Fisher Scientific). In all cases, nuclei were counterstained by incubation with DAPI (1.5 μg∙mL^− 1^; Sigma) for 5 min. Stained cells were analysed under a fluorescence microscope.

### qRT-PCR

Total RNA from frozen cell pellets was extracted using the High Pure RNA Isolation Kit (Roche, Switzerland). Following quantification in a NanoVue™ Plus spectrophotometer (GE Healthcare, USA), 1 μg of RNA was converted to cDNA using the High-Capacity cDNA Reverse Transcriptase Kit (Life Technologies). Reactions were run in triplicate using NZYSpeedy qPCR Green Master Mix, ROX plus (NZYTech, Portugal), and primers specific for *OCT4*, *NANOG*, *SOX1*, *T/BRACHYURY* and *SOX17* in a StepOne Plus Real-Time PCR System (Thermo Fisher Scientific). C_T_ values for each condition were normalised against the corresponding expression of the housekeeping gene glyceraldehyde-3-phosphate dehydrogenase (*GAPDH*), generating ΔC_T_. RNA levels were computed as 2^–ΔCT^.

### Differentiation potential assays

The differentiation potential of hiPSCs was evaluated through the EB assay. Following harvesting of cells from the PBS MINI 0.1, they were replated on 6-well ultra-low attachment tissue culture plates (Corning) in mTeSR1 supplemented with Y-27632. After 24 h, the medium was changed to EB medium, containing KnockOut (KO)-Dulbecco’s Modified Eagle’s Medium (DMEM) supplemented with 20% foetal bovine serum (FBS), 1% (V/V) Minimum Essential Medium (MEM) non-essential amino acids, 1 mmol∙L^− 1^ L-glutamine and 1% (V/V) penicillin/streptomycin (all from Thermo Fisher Scientific), which was refreshed every 2 days during 4 weeks. After differentiation, the EBs were dissociated using 0.05% Trypsin-EDTA (Thermo Fisher Scientific) and plated on a 24-well tissue culture plate coated with 15 μg∙mL^− 1^ poly-ornithine (Sigma) and 20 μg∙mL^− 1^ laminin (Sigma). EB medium was changed every 2 days during 1 week, after which the cells were stained for TUJ1, α-SMA and SOX17. Directed differentiation into both cardiomyocytes and neural progenitors was also performed. Cardiac differentiation was performed through temporal modulation of the WNT signalling pathway [39, 40]. Briefly, hiPSCs were plated on a 12-well Matrigel-coated tissue culture plate at a density of 100,000 cells∙cm^− 2^. After reaching 100% confluence, the medium was changed to RPMI/B27-ins (RPMI 1640 medium (Thermo Fisher Scientific), supplemented with 1× B-27 minus insulin (Thermo Fisher Scientific)), with 6 μmol∙L^− 1^ of CHIR99021 (StemGent). After 24 h, the medium was changed to RPMI/B27-ins. At day 3, a half-medium change was performed with RPMI/B27-ins supplemented with IWP-4 (StemGent) to a final concentration of 5 μmol∙L^− 1^, which was removed with the medium change at day 5. Starting from day 7, the medium was changed every 3 days to RPMI/B27 (RPMI 1640 medium supplemented with 1× B-27 (Thermo Fisher Scientific)) until day 15, when the cells were fixed and stained for cTNT. Neural induction was performed through the dual-SMAD inhibition protocol [41, 42]. Briefly, hiPSCs were plated on a 24-well Matrigel-coated tissue culture plate at a density of 50,000 cells∙cm^− 2^. After reaching 100% confluence, the medium was changed to N2B27, a 1:1 mixture of DMEM/F12 and Neurobasal medium with 1× N2 and 1× B-27, respectively (all from Thermo Fisher Scientific), supplemented with 10 μmol∙L^− 1^ of SB431542 (Sigma) and 100 nmol∙L^− 1^ of LDN193189 (StemGent). Complete medium was refreshed daily for 12 days, after which the cells were fixed and stained for PAX6 and NESTIN.

### Statistical analyses

At least three biological replicates were performed for each experiment. Data are expressed as the mean ± standard error of the mean (SEM). Statistical analyses were performed using GraphPad Prism 6 (GraphPad Software, USA). Statistical significance was determined by two-way ANOVA followed by Tukey’s multiple comparisons test. Differences were considered statistically significant at *p < 0.05, **p < 0.01 and ***p < 0.001.

## Supplementary information

**Additional file 1: Figure S1.** Statistical analysis of hiPSC growth curves. A set of two different culture conditions is presented in each graph: **a** mTeSR1 vs. mTeSR1 + DS; **b** mTeSR3D vs mTeSR3D + DS; **c** mTeSR1 + DS vs mTeSR3D + DS. Differences between conditions, at a given day, were considered statistically significant at **p < 0.01 and ***p < 0.001. **Figure S2.** Flow cytometry analysis of hiPSCs cultured in the VWBR under the different conditions tested: **a** mTeSR1; **b** Gibco hiPSC line, mTeSR1; **c** mTeSR3D; **d** mTeSR1 + DS; **e** mTeSR3D + DS. For each condition, representative images of a 2D dot plot showing population gating and histograms of OCT4, TRA-1-60 and SSEA-1 analyses, including negative controls (grey) are shown. **Figure S3.** Number of hiPSC aggregates at day 1 under the different culture conditions tested. Samples (700 μL) harvested from the VWBR at day 1 were placed in a 24-well tissue culture plate and, using an optical microscope, pictures were taken, capturing all the aggregates present. Images were then analysed to count the total number of aggregates in the sample, which allowed to estimate the number of aggregates in the whole vessel. A total of two samples from two different runs were analysed for each condition. **Figure S4.** Cell viability analysis. hiPSC aggregates cultured for **a** 3 and **b, c** 7 days in the VWBR, with mTeSR1 + DS, were harvested, incubated with calcein AM (2 μM) and ethidium homodimer (4 μM; Sigma) for 30 min and visualised using a confocal microscope. Maximum intensity projections are shown (scale bars = 100 μm). Aggregates at day 7 were also dissociated, stained with the LIVE/DEAD Fixable Far Red Dead Cell Stain Kit (ThermoFisher), according to the manufacturer instructions and analysed by flow cytometry. Representative images of **d** a 2D dot plot showing population gating and **e** an histogram of a sample analysis (orange), including the control (a 50/50 mix of live cells and dead cells obtained by thermal shock) (grey). Three independent samples were analysed and percentage of live cells is shown as mean ± SEM. **Figure S5** Negative control of the antibody stainings performed in Fig. 5c. Differentiated cells were stained for OCT4 (secondary antibody: goat anti-mouse IgG-AlexaFluor 546), which is not present in these cells (scale bar = 50 μm).

## Data Availability

The datasets used and/or analysed during the current study are available from the corresponding author on reasonable request.

## Abbreviations

bFGF: Basic fibroblast growth factor
cGMP: Current Good Manufacturing Practice
DMEM: Dulbecco’s Modified Eagle’s Medium
DoE: Design of Experiments
DPBS: Dulbecco’s phosphate-buffered saline
DS: Dextran sulfate
EB: Embryoid body
GAPDH: Glyceraldehyde-3-phosphate dehydrogenase
hESC: Human embryonic stem cell
hiPSC: Human induced pluripotent stem cell
hPSC: Human pluripotent stem cell
KO: KnockOut
MEM: Minimum Essential Medium
OXPHOS: Oxidative phosphorylation
qRT-PCR: Quantitative real-time polymerase chain reaction
SEM: Standard error of the mean
TGFβ: Transforming growth factor β
VWBR: Vertical-Wheel bioreactor
