# Supplementary material for: Strategies for the expansion of human induced pluripotent stem cells as aggregates in single-use Vertical-Wheel™ bioreactors
Source: J Biol Eng. 2019 Sep 14;13:74. doi: 10.1186/s13036-019-0204-1 (PMC6744632; doi:10.1186/s13036-019-0204-1)
Supplement: Supplementary file 1 — Additional file 1: Figure S1. Statistical analysis of hiPSC growth curves. A set of two different culture conditions is presented in each graph: a mTeSR1 vs. mTeSR1 + DS; b mTeSR3D vs mTeSR3D + DS; c mTeSR1 + DS vs mTeSR3D + DS. Differences between conditions, at a given day, were considered statistically significant at **p < 0.01 and ***p < 0.001. Figure S2. Flow cytometry analysis of hiPSCs cultured in the VWBR under the different conditions tested: a mTeSR1; b Gibco hiPSC line, mTeSR1; c mTeSR3D; d mTeSR1 + DS; e mTeSR3D + DS. For each condition, representative images of a 2D dot plot showing population gating and histograms of OCT4, TRA-1-60 and SSEA-1 analyses, including negative controls (grey) are shown. Figure S3. Number of hiPSC aggregates at day 1 under the different culture conditions tested. Samples (700 μL) harvested from the VWBR at day 1 were placed in a 24-well tissue culture plate and, using an optical microscope, pictures were taken, capturing all the aggregates present. Images were then analysed to count the total number of aggregates in the sample, which allowed to estimate the number of aggregates in the whole vessel. A total of two samples from two different runs were analysed for each condition. Figure S4. Cell viability analysis. hiPSC aggregates cultured for a 3 and b, c 7 days in the VWBR, with mTeSR1 + DS, were harvested, incubated with calcein AM (2 μM) and ethidium homodimer (4 μM; Sigma) for 30 min and visualised using a confocal microscope. Maximum intensity projections are shown (scale bars = 100 μm). Aggregates at day 7 were also dissociated, stained with the LIVE/DEAD Fixable Far Red Dead Cell Stain Kit (ThermoFisher), according to the manufacturer instructions and analysed by flow cytometry. Representative images of d a 2D dot plot showing population gating and e an histogram of a sample analysis (orange), including the control (a 50/50 mix of live cells and dead cells obtained by thermal shock) (grey). Three independent samples we [file 13036_2019_204_MOESM1_ESM.docx]

Supporting Information

for

Strategies for the Expansion of Human Induced Pluripotent Stem Cells as Aggregates
in Single-Use Vertical-Wheel^TM^ Bioreactors

Diogo E.S. Nogueira^1,2^, Carlos A.V. Rodrigues^1,2^, Marta S. Carvalho^1,2^, Cláudia C. Miranda^1,2^,
Yas Hashimura^3^, Sunghoon Jung^3^, Brian Lee^3^ and Joaquim M.S. Cabral^1,2^

^1^Department of Bioengineering and iBB – Institute for Bioengineering and Biosciences, Instituto Superior Técnico, Universidade de Lisboa, Lisboa, Portugal

^2^The Discoveries Centre for Regenerative and Precision Medicine, Lisbon Campus, Instituto Superior Técnico, Universidade de Lisboa, Lisboa, Portugal

^3^PBS Biotech, Camarillo, CA, USA

# Corresponding author:

Carlos A.V. Rodrigues

Address: Institute for Bioengineering and Biosciences, Instituto Superior Técnico, Av. Rovisco Pais, 1049-001, Lisboa, Portugal

E-mail: carlos.rodrigues@tecnico.ulisboa.pt

Phone: +351 21 040 70 51


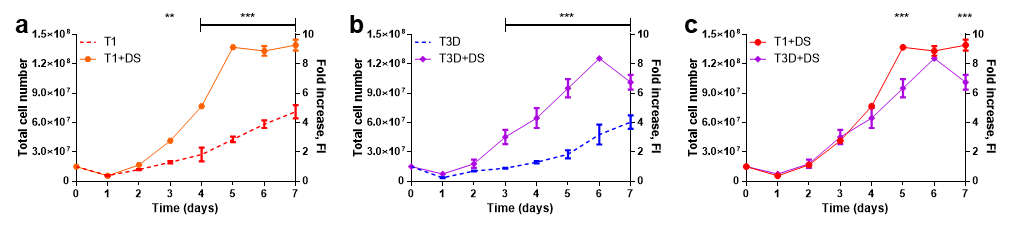


**Figure S1.** Statistical analysis of hiPSC growth curves. A set of two different culture conditions is presented in each graph: **a** mTeSR1 vs mTeSR1+DS; **b** mTeSR3D vs mTeSR3D+DS; **c** mTeSR1+DS vs mTeSR3D+DS. Differences between conditions, at a given day, were considered statistically significant at ***p*<0.01 and ****p*<0.001.


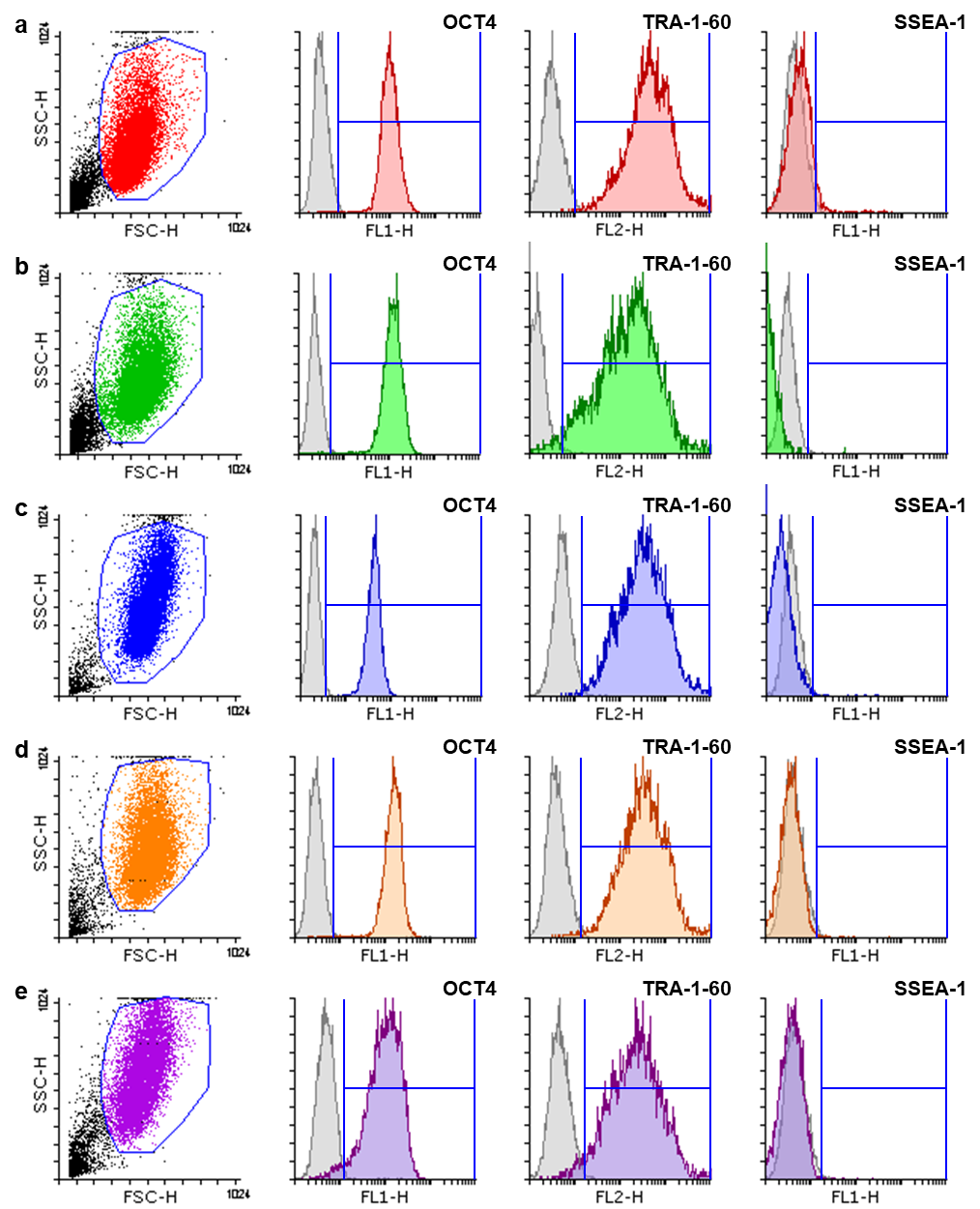


**Figure S2.** Flow cytometry analysis of hiPSCs cultured in the VWBR under the different conditions tested: **a** mTeSR1; **b** Gibco hiPSC line, mTeSR1; **c** mTeSR3D; **d** mTeSR1+DS; **e** mTeSR3D+DS. For each condition, representative images of a 2D dot plot showing population gating and histograms of OCT4, TRA-1-60 and SSEA-1 analyses, including negative controls (grey) are shown.


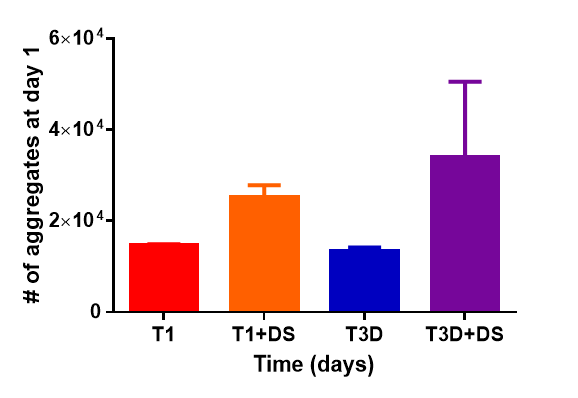


**Figure S3.** Number of hiPSC aggregates at day 1 under the different culture conditions tested. Samples (700 μL) harvested from the VWBR at day 1 were placed in a 24-well tissue culture plate and, using an optical microscope, pictures were taken, capturing all the aggregates present. Images were then analysed to count the total number of aggregates in the sample, which allowed to estimate the number of aggregates in the whole vessel. A total of two samples from two different runs were analysed for each condition.


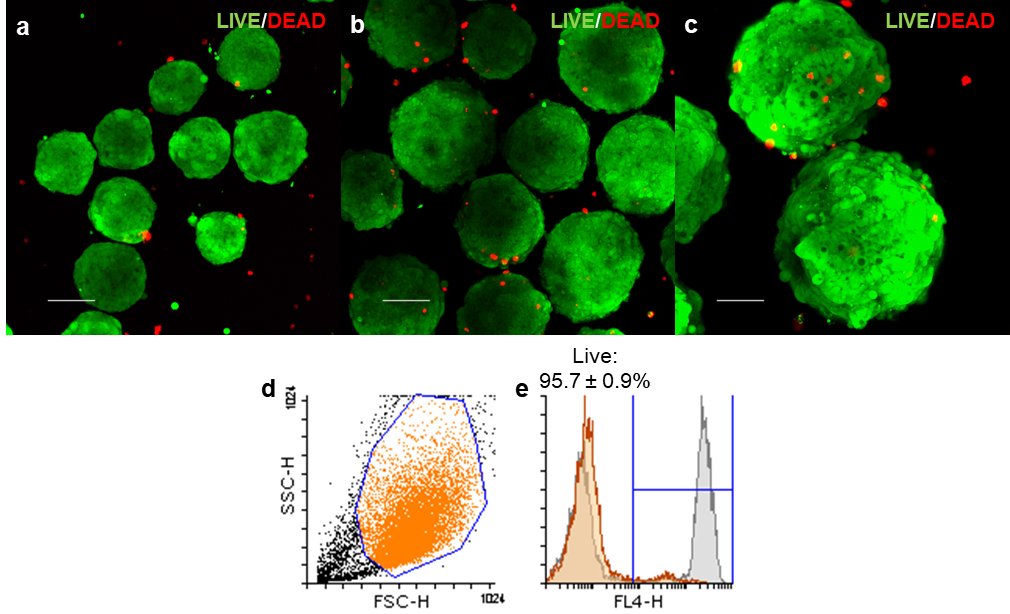


**Figure S4.** Cell viability analysis. hiPSC aggregates cultured for **a** 3 and **b, c** 7 days in the VWBR, with mTeSR1+DS, were harvested, incubated with calcein AM (2 μM) and ethidium homodimer (4 μM; Sigma) for 30 min and visualised using a confocal microscope. Maximum intensity projections are shown (scale bars = 100 μm). Aggregates at day 7 were also dissociated, stained with the LIVE/DEAD Fixable Far Red Dead Cell Stain Kit (ThermoFisher), according to the manufacturer instructions and analysed by flow cytometry. Representative images of **d** a 2D dot plot showing population gating and **e** a histogram of a sample analysis (orange), including the positive control (a 50/50 mix of live cells and dead cells obtained by thermal shock) (grey). Three independent samples were analysed and percentage of live cells is shown as mean ± SEM.


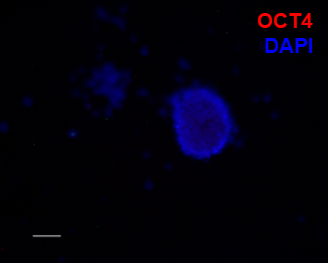


**Figure S5.** Negative control of the antibody stainings performed in Fig. 5c. Differentiated cells were stained for OCT4 (secondary antibody: goat anti-mouse IgG-AlexaFluor 546), which is not present in these cells (scale bar = 50 μm).
